# A rare case of clear cell Hidradenocarcinoma presenting with recurrent pleural and cardiac effusion

**DOI:** 10.3332/ecancer.2025.1969

**Published:** 2025-08-19

**Authors:** Camilla Engelsmann, Gitte Wooler, Vladimira Horvat, Shailesh Balasaheb Kolekar

**Affiliations:** 1 Department of Medicine, Zealand University Hospital, Roskilde 4000, Denmark; 2 Department of Histopathology, Copenhagen University Hospital – Herlev and Gentofte, 2730, Denmark; 3 Department of Clinical Medicine, University of Copenhagen, Copenhagen 2000, Denmark; 4 Department of Oncology, Roskilde University Hospital, Roskilde 4000, Denmark; 5 Copenhagen University, Copenhagen 2000, Denmark

**Keywords:** clear cell hidradenocarcinoma, hidradenocarcinoma, sweat gland tumour, skin tumours, metastasis

## Abstract

Clear cell hidradenocarcinoma (HAC) is a rare and aggressive malignant tumour originating from eccrine sweat glands, accounting for only 0.001% of all tumours. HAC primarily occurs in the head and neck region, with a high propensity for local recurrence and distant metastases. This case report details the unusual presentation of a 66-year-old male with a history of myocardial infarction, hypertension and a significant smoking history, who presented with abdominal pain and progressive shortness of breath. Diagnostic imaging revealed pleural and pericardial effusion and initial workup, including biopsies from pleura, skin and lymph nodes, was inconclusive. Subsequent histopathological examination suggested a diagnosis of HAC with metastases to the pleura, pericardium, lymph nodes, bones and subcutaneous tissues. Despite aggressive diagnostic efforts, the patient succumbed to the disease before chemotherapy could be initiated. This case underscores the diagnostic challenges of HAC, particularly with its atypical presentation and rare metastatic sites, such as the pleura and pericardium. The report emphasises the need for awareness of this rare malignancy and its potential for rapid, fatal progression.

## Introduction

Clear cell hidradenocarcinoma (HAC) is a rare and aggressive eccrine tumour of the sweat glands. It accounts for 0.001% of all tumours [1–4] and 6% of eccrine tumours [2, 5, 6]. Studies suggest a higher prevalence in males compared to females [1, 2, 7]. HAC most commonly debuts around the age of 50–70 years; however, cases in children have been noted [4, 5, 7, 8]. HAC is most commonly found in the head and neck area, but is rarely, seen on the trunk, gluteal area and extremities [1, 2, 4, 7, 9]. It usually arises de novo and rarely from preexisting hidradenomas [10]. Distant metastases are seen in 39% of cases [7]. Most commonly, HAC metastasises to the lymph nodes, but metastases have been seen in multiple organs such as lungs, pleura, liver, bone, including vertebrae and bone marrow [1, 3–5, 8, 9]. Post-surgical recurrence is common and is seen in 60 % of cases. Five-year post-surgical survival is only 30% [3–6].

We present a case of multiple metastases of HAC with a rare presentation where diagnosis proved difficult, and the outcome was fatal.

## History

A 66-year-old male with a history of myocardial infarction, hypertension and a smoking history of 25–30 pack-years, presented to his primary care physician with abdominal pain. He later admitted to 3 weeks of progressive shortness of breath.

An ultrasound of the abdomen showed no intraabdominal pathology but found pleural effusion. The patient was referred for a chest X-ray, which showed fluid in both the pericardium and pleura bilaterally. Computed tomography (CT) of the thorax and abdomen showed 3 cm pericardial effusion, pleural effusion and was suspicious of malignant mesothelioma with metastases to the subcutis of the truncal skin and left pectoral muscle.

**Figure 1.**
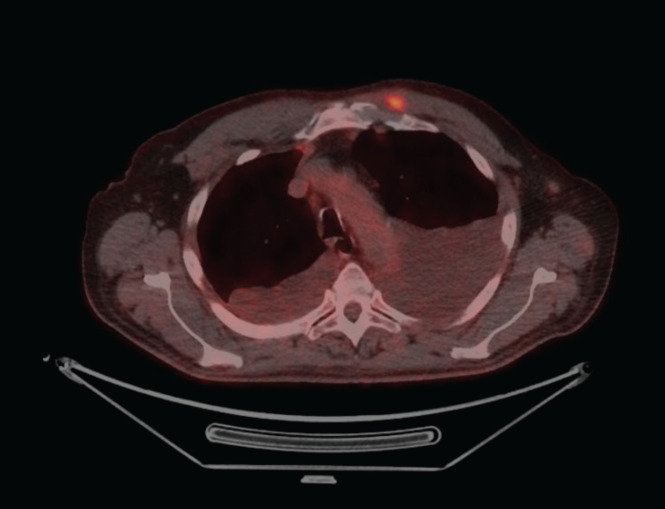
PET- CT with FDG uptake in a metastasis of the left pectoral muscle.

**Figure 2.**
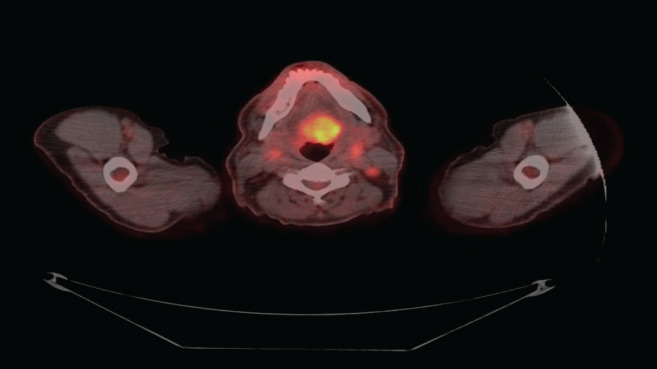
FDG PET increased uptake of FDG in the root of the tongue.

**Figure 3.**
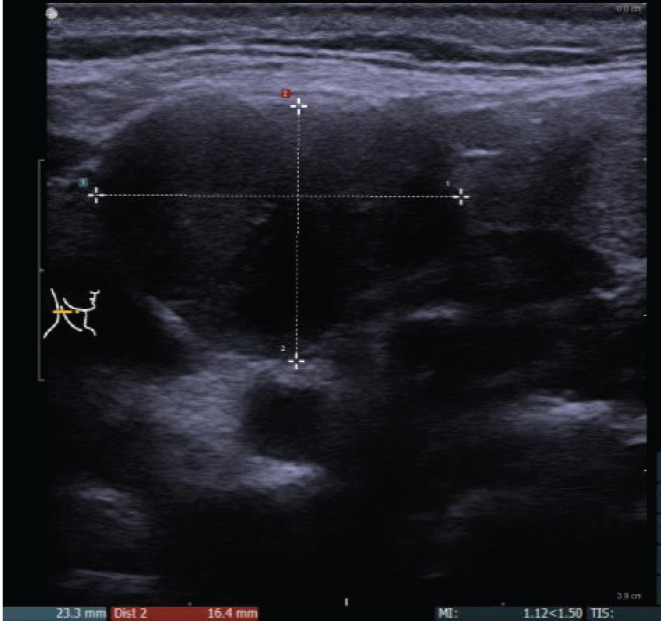
Hypoechoic conglomerate at lvl. 2–3 on the right side of the neck suggesting lymph node conglomerate.

Later, a positron emission tomography (PET)-CT scan showed increased ^18^Fluor-Deoxy-Glucose uptake in the rod of the tongue, lymph nodes on the neck, tumours in the truncal subcutis and left pectoral muscles. The PET-CT also suggested metastasising to the bones, flank and gluteal area (Figures 1 and 2). Initial tests also included diagnostic examinations of the pleural fluid and a pleural biopsy and the patient was referred to the department of Otorhinolaryngology Head & Neck Surgery, where a laryngoscopy was performed with biopsies from the base of the tongue and biopsies from cervical lymph nodes (Figure 3). Mesothelial hyperplasia was found in the pleural fluid and the histopathological patterns from the lymph node biopsy suggested an adnexal tumour, presumed to be HAC; however, it could not rule out squamous cell cancer or a possible metastasis.

A magnetic resonance scan showed tumour growth into the mastication muscles. The patient then underwent an oropharyngoscopy with multiple re-biopsies from the tongue, never finding signs of malignancy.

**Figure 4.**
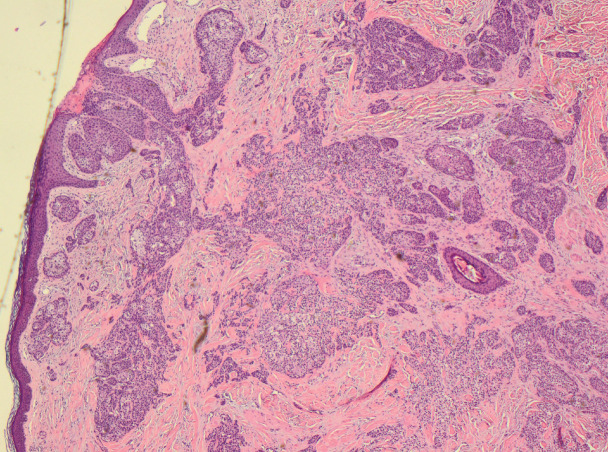
Skintumour in close contact with an adnexal structure.

**Figure 5.**
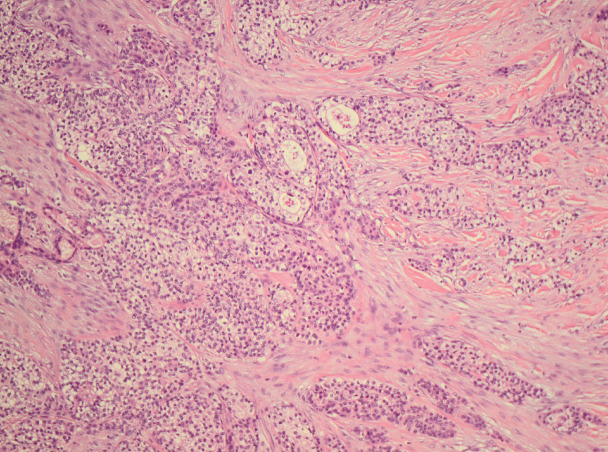
Area of tubular structure.

**Figure 6.**
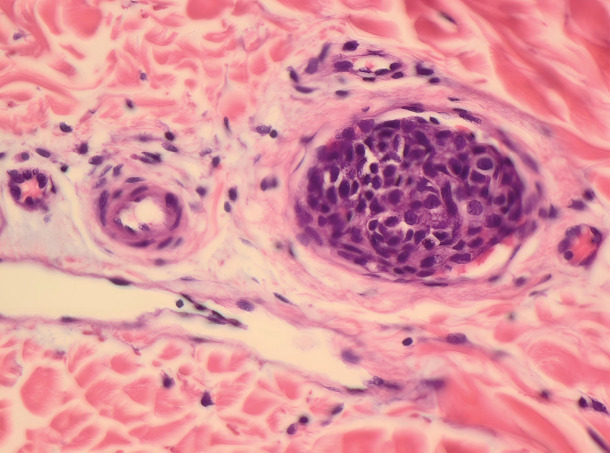
Area of vascular invasion.

Later, a biopsy from a tumour in the pectoral muscle was performed; however, it was not possible to get a sufficient amount of tissue for diagnosis. A video-assisted thoracoscopy biopsy of the right parietal pleura was performed with no clear diagnosis. At the same time, a re-excision of the tumour in the pectoral muscle was performed and showed carcinoma metastasis from an unknown tumour. Afterward, the patient was referred to a dermatologist for excision of the truncal subcutaneous tumour (Figures 4–6), and the pathological diagnosis suggested a HAC in the skin, denying suggestions of metastasis originating from the lung or pleura or clear cell carcinoma of the tongue, as first believed. Later histopathological patterns from another lymph node biopsy supported this diagnosis.

Because of fast-developing pleural- and pericardial fluid, the patient had more than 14 pleurocenteses and multiple pericardiocentesis during diagnostic evaluation.

The patient was referred to chemotherapy at Rigshospitalet, Copenhagen Denmark, but died less than 3 months after initial referral before receiving any treatment.

## Discussion

Clear cell hidradenocarcinoma is a rare and aggressive tumour, that arises from the intradermal duct of eccrine sweat glands. Microscopical analysis of HAC varies, but most commonly it shows a dermal-based neoplasm with ductal structures, intracellular lumen formation, variable clear cell changes and an infiltrative growth pattern [7].

Usually, HAC originates in the head and neck area. HAC is known to metastasise to lymph nodes and visceral organs [1, 3, 5, 8, 9]. Usually, patients present with a tumour of the skin in the early stages [9], and no other symptoms. In this case, the patient presented with abdominal pain and shortness of breath, and later diagnostic imaging showed metastases to multiple organs.

Initial biopsies from both the pectoral tumour, lymph nodes on the neck of the tongue either showed no malignancy or a metastasis of an unknown primary tumour. Later skin excision from the left pectoral area showed carcinoma with an expansive front, ductal structures, cells with intracellular laminae, high levels of mitosis, cells with light eosinophilic cytoplasm and clear cells, suggesting HAC as the primary diagnosis (Figures 4–6).

The patient presented with metastasis to lymph nodes, visceral- and pleural metastases and multiple skin tumours. Only a few cases of metastases to the pleural wall have been described in English literature [3, 5, 8], but multiple cases describe metastases to the lungs and lymph nodes [1, 3–5, 9].

At first, the conclusion of the multi-disciplinary team was that the patient suffered from clear cell adenocarcinoma (CCC) of the tongue. CCC of the tongue is a rare tumour that has been known to metastasise to the lungs [11] and rarely to the skin [12]. However, in this case, no biopsies from the tongue or other metastases suggested CCC, while two biopsies from different locations suggested other differential diagnosis: HAC.

## Conclusion

The case presents a rare and aggressive form of HAC with unusual metastatic spread and provides valuable insight into the diagnostic challenges. By comparing HAC with other conditions like CCC, the case highlights the complexity of diagnosing rare tumours.

## List of abbreviations

HAC, Clear cell hidradenocarcinoma; CT, Computed tomography; PET, Positron emission tomography; SCC, Squamous cell cancer; MR, Magnetic resonance scan; CCC, Clear cell adenocarcinoma.

## Conflicts of interest

No conflict of interest.

## Funding

No funding was received for this study.

## Informed consent

Informed consent was obtained and an informed consent form was filled out.

## Author contributions

All authors have contributed to this article.
